# Structural and Nutritional Peculiarities Related to Lifespan Differences on Four *Lopesia* Induced Bivalve-Shaped Galls on the Single Super-Host *Mimosa gemmulata*

**DOI:** 10.3389/fpls.2021.660557

**Published:** 2021-05-17

**Authors:** Elaine C. Costa, Denis C. Oliveira, Dayse K. L. Ferreira, Rosy M. S. Isaias

**Affiliations:** ^1^Departamento de Botânica, Instituto de Ciências Biológicas, Universidade Federal de Minas Gerais, Belo Horizonte, Brazil; ^2^Instituto de Biologia, Universidade Federal de Uberlândia, Uberlândia, Brazil

**Keywords:** cell walls, gall anatomy, hemicelluloses, histochemistry, immunocytochemistry, *Lopesia* galls

## Abstract

Super-host plants are elegant models to evaluate the peculiarities of gall structural and nutritional profiles due to the stimuli of distinct gall inducers in temporal and spatial perspectives. Galls induced by congeneric insects, *Lopesia* spp. (Diptera, Cecidomyiidae) on the same host plant, *Mimosa gemmulata* Barneby (Fabaceae) were analyzed to estimate if variations of 1 or 2 months in gall lifespans may result in differences over the accumulation of nutritional resources, and their compartmentalization both in cell walls and protoplasm. *Mimosa gemmulata* hosts four *Lopesia*-induced galls: the lenticular bivalve-shaped gall (LG) with a 2-month life cycle, the brown lanceolate bivalve-shaped gall (BLG) and the green lanceolate bivalve-shaped gall (GLG) with 3 month-life cycles, and the globoid bivalve-shaped gall (GG) with a 4 month-life cycle. The comparisons among the four *Lopesia* galls, using anatomical, histometric, histochemical, and immunocytochemical tools, have demonstrated that the longest lifespan of the GG related to its highest increment in structural and nutritional traits compared with the LG, GLG, and BLG. The differences among the tissue stratification and cell wall thickness of the galls with the 2-month and the 3-month lifespans were subtle. However, the GG had thicker cell walls and higher stratification of the common storage tissue, schlerenchymatic layers and typical nutritive tissue than the other three gall morphospecies. The higher tissue thickness of the GG was followed by the formation of a bidirectional gradient of carbohydrates in the protoplasm, and the detection of xyloglucans in cell walls. Current data supported the presumption that the longest the lifespan, the highest the impact over the structural and nutritional metabolism of the *Lopesia* galls associated to *M. gemmulata*.

## Introduction

The gall lifespans depend on structural, cytological, and chemical traits on the host plant cells stimulated by the associated galling organisms (Mani, 1964; Oliveira et al., 2010, 2016; Jorge et al., 2018; Ferreira et al., 2019; Chen et al., 2020). Any changes in the galling organism behavior may lead to the disruption of the gall life cycle (Rezende et al., 2019) and compromise gall developmental stages. In mature galls, the specialized tissues are established and organized in specific compartments (Bragança et al., 2017), which store energy-rich molecules that support the galling organism nutritional requirements (Mani, 1964; Ferreira et al., 2017). Cecidomyiidae galls, for instance, can have two types of storage tissues: the common storage tissue and the typical nutritive tissue, which are commonly spatially separated by schlerenchymatic layers (Meyer and Maresquelle, 1983; Bronner, 1992; Rohfritsch, 1992). The common storage tissue is located in the gall outer tissue compartment, while the typical nutritive tissue is located in the gall inner tissue compartment, which is in contact with the larval chamber (Bragança et al., 2017).

The cells of the common storage tissue have inconspicuous nuclei, large vacuoles, thin cytoplasm, and accumulate energetic metabolites related to gall growth and metabolism. The storage tissue also supports the nutritive tissue by cell-to-cell translocation of solutes (Moura et al., 2008; Oliveira et al., 2010; Ferreira and Isaias, 2014). The cells of the typical nutritive tissue have conspicuous nuclei, fragmented vacuoles, and dense protoplasm, which accumulate energetic metabolites related to the nutrition of the gall inducer (Bronner, 1992; Oliveira et al., 2010, 2011a; Ferreira et al., 2015). The additional accumulation of carbohydrates in cell walls have been recently evaluated in nematode-induced galls (Ferreira et al., 2020) and in insect-galls (Bragança et al., 2020a), with peculiarities regarding the type and distribution of hemicellulose epitopes. The hemicelluloses, xyloglucans and heteromannans, integrate the primary or secondary cell walls, may regulate cell expansion (Cosgrove, 2016, 2018), and have also been related to the galling organism maintenance (Bragança et al., 2020a; Ferreira et al., 2020). In an overall analysis, the storage and nutritive tissues accumulate different metabolites in Cecidomyiidae galls (Bronner, 1992; Moura et al., 2008; Oliveira et al., 2010) but can perform specific functions in the diverse host plant-galling insect systems (Amorim et al., 2017; Bragança et al., 2017). Accordingly, the longer the galling insect stay inside the gall, the higher the gall demand is (Carneiro et al., 2017), therefore, the long-life cycle of the galling insect may determine more complex gall structural profiles (Gonçalves et al., 2005). Such presumption may be analyzed in the nutritional perspective, and the accumulation of energetic resources in gall storage tissues can be higher, the longer the gall lifespan is, which may be elegantly evaluated in super-host plants with several associated galling-insects (Formiga et al., 2014; Amorim et al., 2017; Bragança et al., 2020a; Costa et al., submitted).

Currently, we use anatomical, histometric, histochemical, and immunocytochemical tools to evaluate the structural and nutritional profiles of four bivalve-shaped galls induced by four undescribed new species of *Lopesia* (Rübsaamen, 1908) (Diptera-Cecidomyiidae; Maia and Carvalho-Fernandes personal communication) in temporal and spatial perspectives on the super-host *Mimosa gemmulata* Barneby (Fabaceae). These galls have distinct lifespans and their gall-inducing *Lopesia* species have multivoltine life cycles whose durations vary in 2-, 3-, or 4-months (Costa et al., submitted). We expect that variations of 1 or 2 months in gall lifespans may result in differences over the accumulation of nutritional resources, and their compartmentalization both in cell walls and protoplasm. The following questions are addressed: (1) are there distinct peculiarities in the structural profiles among the four *Lopesia* galls regarding tissue compartments? And (2) may the nutritional profiles of the four *Lopesia* galls vary in response to the 1–2-month-temporal distinction?

## Materials and Methods

### The Lifespans of *Lopesia* Galls on *M. gemmulata*

The four *Lopesia* galls induced on *M. gemmulata* pinna-rachis are the lenticular bivalve-shaped gall (LG), the green lanceolate bivalve-shaped gall (GLG), the brown lanceolate bivalve-shaped gall (BLG), and the globoid bivalve-shaped gall (GG). The four *Lopesia* spp. have multivoltine life cycles and each bivalve-shaped gall have distinct developmental times, with the maturation as the longest stage of development. The LG has six life cycles a year, the BLG and GLG have four life cycles a year, and the GG has three cycles a year (Costa et al., submitted). The LG has a 2-month life cycle, with the maturation stage lasting ≅ 30–45 days. The BLG and GLG have 3 month-life cycles, with the maturation stage lasting ≅ 45–60 days. The GG has a 4 month-life cycle, with the maturation stage lasting ≅ 60–75 days.

### Structural Analysis

Samples of the non-galled pinna-rachis (control) and of the LG, GLG, BLG, and GG (mature galls with live larvae) were collected (*n* = 5 for each gall system) from individuals of *M. gemmulata* (*n* = 5) in a Cerrado area located at Serra Geral, municipality of Caetité, state of Bahia, Brazil (14°04′36.8″S, 42°29′59″W) on March 2019. For anatomical and immunocytochemical analyses, a set of fragments of the pinna-rachis, LG, GLG, BLG, and GG (*n* = 8 for each gall morphospecies) were fixed in 2.5% glutaraldehyde and 4.5% formaldehyde in 0.1 mol.L^–1^ (Karnovsky, 1965, modified to pH 7.2 phosphate buffer), for 48 h at room temperature. The fixed fragments were dehydrated in an ethanol series and embedded in Paraplast^®^ (Kraus and Arduin, 1997). The sections (12 μm) were obtained in a rotary microtome (Leica^®^ BIOCUT 2035), deparaffinized in butyl acetate, and hydrated in an ethanol series (Kraus and Arduin, 1997). The sections (*n* = 5 for each category) were stained in Astra blue and safranin (9:1, v/v) (Bukatsch, 1972, modified to 0.5%) dehydrated in an ethanol-butyl acetate series (Kraus and Arduin, 1997), and mounted using colorless varnish Acrilex^®^ (Paiva et al., 2006). A second set of fragments of the pinna-rachis and of the four mature *Lopesia* galls (*n* = 17 for each gall morphospecies) was used for histochemical analysis. The histological slides were analyzed and photographed under a light microscope (Leica^®^ DM500) with a coupled digital camera (Leica^®^ ICC50 HD).

### Histometric Analysis

The thickness of the common storage tissue, schlerenchymatic layers, and typical nutritive tissue, as well as the respective cell walls, were measured in the LG, BLG, GLG, and GG (*n* = 5 galls, one section per gall, 5 measurement fields per section, totalizing 25 measurements by tissue for each gall morphospecies). The data were compared using one-way ANOVA followed by Tukey’s test, using α = 0.05. The tests were performed with SigmaStat^®^ (Systat Software, Inc., Chicago, Illinois) and the graphics were done with GraphPad prism 5.0^®^.

### Histochemical Analysis

Free-handmade sections from fresh samples of the pinna-rachis, LG, GLG, BLG, and GG (*n* = 7 for each gall morphospecies) and Paraplast^®^ embedded sections obtained in a rotary microtome were submitted to histochemical analyses. Starch grains were detected with Lugol’s reagent (1% potassium iodine-iodide solution) for 5 min (Johansen, 1940). Reducing sugars were detected by Fehling’s reagent (Solution A: 7.9% copper sulfate, and solution B: 34.6% sodium potassium tartrate and 1% sodium hydroxide) heated to pre-boiling temperature (Sass, 1951). Proteins were detected by 0.1% bromophenol blue in a saturated solution of 10% magnesium chloride in ethanol during 15 min, washed in 0.5% acetic acid in water during 20 min, and water for 3 min (Mazia et al., 1953). Lipids were detected with a saturated solution of Sudan Red B in 70 GL ethanol during 5 min (Brundett et al., 1991). Black sections were used as controls. The sections were analyzed and photographed under a light microscope (Leica^®^ DM500) coupled to a digital camera (Leica^®^ ICC50 HD).

### Immunocytochemical Analysis

The detection of hemicelluloses was performed in the sections of the pinna-rachis, LG, GLG, BLG, and GG (*n* = 3 for each category) obtained in a rotary microtome. The sections were pre-incubated in pectate lyase at 10 μg/mL, diluted in 50 mM N-cyclohexyl-3-aminopropane sulfonic acid (CAPS) and 2 mM CaCl_2_ buffer, pH 10, for 2 h at room temperature (Marcus et al., 2008). Afterward, the sections were incubated in the primary monoclonal antibodies (MAbs), LM15 and LM21, diluted in block solution [5% powder milk in phosphate-buffered saline-PBS) 0.1 mol L^1^, pH 7.2 (1:5, *w*/*v*)] for the labeling of the epitopes of xyloglucans (Marcus et al., 2008) and heteromannans (Marcus et al., 2010), respectively, for 90 min in the darkness. The sections were washed in PBS and incubated in the secondary antibody anti-rat IgG linked to FITC, diluted in 5% powder milk/PBS (1:100, *w*/*v*), for 90 min in darkness. The slides were mounted in 50% glycerin, analyzed and photographed under a fluorescence microscope (Leica^®^ DM 2500 LED), with blue excitation light (450--490 nm) and green emission light (515 nm), coupled to a digital camera (Leica^®^ DFC 7000T). The immunocytochemical images were submitted to intensity measurement using ImageJ version 1.51k^1^. The fluorescence intensities of the epitopes of hemicelluloses were evaluated by grayscale methodology (Gy = Gray value) with triplicate analysis for each tissue. After the measurements, we proposed the following categories: (−) negative (= 0 Gy values); (+) weak (10–20 Gy values); (++) moderate (21–39.99 Gy values); and (+++) intense (≥40 Gy values).

## Results

### Non-galled Pinna-Rachis Profile (Control)

**FIGURE 1:**
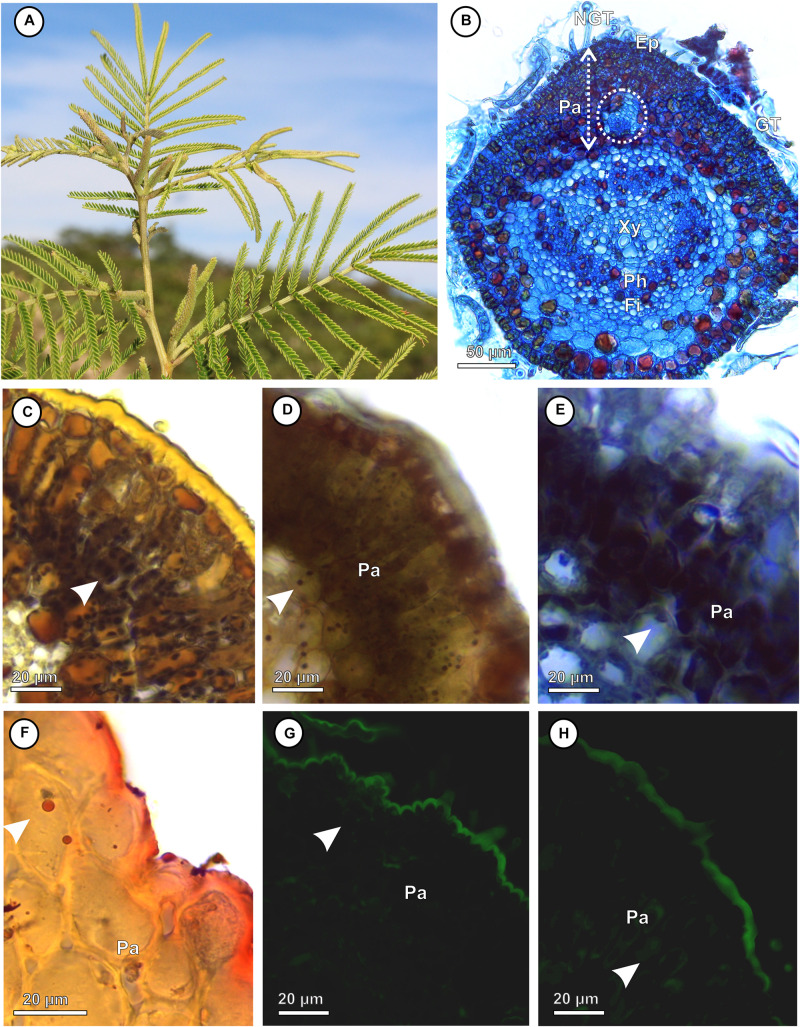
Non-galled pinna-rachis of *Mimosa gemmulata* Barneby (Fabaceae). **(A)** General aspect of non-galled leaves. **(B–H)** Transverse sections. **(B)** Anatomical profile. **(C,D)** Immunocytochemical profile. **(C–F)** Histochemical profile. **(C)** Starch grains stained in black (white arrowhead). **(D)** Reducing sugars stained in brown (white arrowhead). **(E)** Proteins stained in blue (white arrowhead). **(F)** Lipidic droplets stained in red (white arrowhead). **(G,H)** Xyloglucans detected by LM15 in cell walls of parenchyma (white arrowhead). **(H)** Heteromannans detected by LM21 in cell walls of parenchyma (white arrowheads). Ep, Epidermis; Fi, fibers; GT, glandular trichomes; NGT, non-glandular trichomes; Pa, parenchyma; Ph, Phloem; Xy, Xylem.

The pinna-rachis of *M. gemmulata* (Figure 1A) has uniseriated epidermis with glandular and non-glandular trichomes (Figure 1B). The adaxial cortical parenchyma is homogeneous with 3–4 cell layers. The vascular tissues have bicollateral arrangement and are surrounded by two layers of pericyclic fibers (Figure 1B). Starch (Figure 1C), reducing sugars (Figure 1D), proteins (Figure 1E), and lipidic droplets (Figure 1F) accumulate in the protoplasm of parenchyma cells. In the pinna-rachis, the epitopes of xyloglucans recognized by LM15 (24 Gy; Figure 1G) and the epitopes of heretomannans recognized by LM21 (30.4 Gy; Figure 1H) are moderately labeled in the parenchyma cell walls.

### Profiles of *Lopesia* Galls

#### Structural Profiles

**FIGURE 2:**
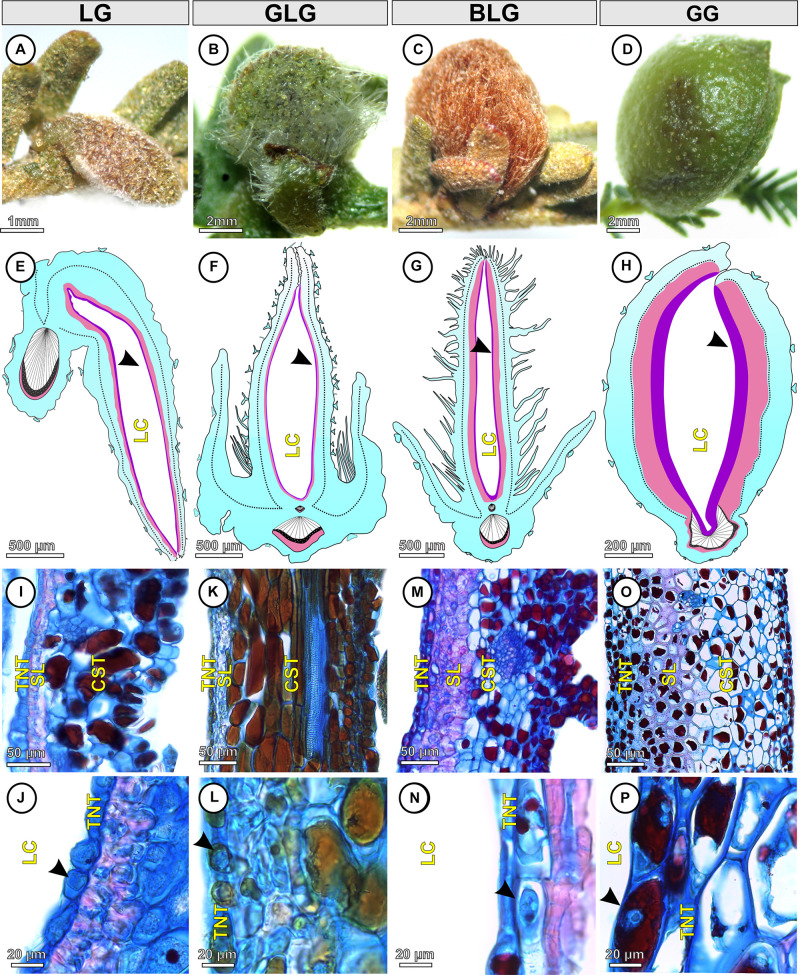
Structural profiles of the four *Lopesia* galls on *Mimosa gemmulata* Barneby (Fabaceae) pinna-rachis in transverse sections. **(A–D)** Macroscopic aspect of the galls. **(A,E)** Lenticular bivalve-shaped gall. **(B,F)** Green lanceolate bivalve-shaped gall. **(C,G)** Brown lanceolate bivalve-shaped gall. **(D,H)** Globoid bivalve-shaped gall. **(E–H)** Diagram of the *Lopesia* galls, evidencing common storage tissues (blue), schlerenchymatic layers (pink) in the outer tissue compartments, and typical nutritive tissues (purple–black arrowheads) in the inner tissue compartments. **(I,J)** Lenticular bivalve-shaped gall, evidencing common storage tissue, schlerenchymatic layer and typical nutritive cells with evident nuclei (black arrowhead). **(K,L)** Green lanceolate bivalve-shaped gall, evidencing common storage tissue, schlerenchymatic layer and typical nutritive cells with evident nuclei (black arrowhead). **(M,N)** Brown lanceolate bivalve-shaped gall, evidencing common storage tissue, schlerenchymatic layer and typical nutritive cells with evident nuclei (black arrowhead). **(O,P)** Globoid bivalve-shaped gall, evidencing common storage tissue, schlerenchymatic layer and typical nutritive cells with evident nuclei (black arrowhead). BLG, brown lanceolate bivalve-shaped gall; CST, common storage tissue; GG, globoid bivalve-shaped gall; GLG, green lanceolate bivalve-shaped gall; LC, larval chamber; LG, lenticular bivalve-shaped gall; SL, schlerenchymatic layer; TNT typical nutritive tissue.

The *Lopesia* galls are green (LG, GLG, and GG) or brown (BLG), isolated, pubescent (Figures 2A–D), and developed by pinna-rachis cell redifferentiation and tissue reorganization. In the four *Lopesia* galls, the epidermis, common storage tissue, vascular tissues, and schlerenchymatic layers form the gall outer compartment and the typical nutritive tissue forms the gall inner compartment (Figures 2E–H). In the LG, the common storage tissue has 4–5 cell layers (Figure 2I), the schlerenchyma has 1–2 layers, and the typical nutritive tissue has 1–2 cell layers (Figure 2J). In the GLG, the common storage tissue has 10–11 cell layers (Figure 2K), the schlerenchyma has 1–2 layers, and the typical nutritive tissue has 1–2 cell layers (Figure 2L). In the BLG, the common storage tissue has 8–9 cell layers (Figure 2M), the schlerenchyma has 4–5 layers, and the typical nutritive tissue has 1–2 cell layers (Figure 2N). In the GG, the common storage tissue has 10–11 cell layers (Figure 2O), the schlerenchyma has 7–8 layers, and the typical nutritive tissue has 5–6 cell layers (Figure 2P).

#### Histometric Profiles

**FIGURE 3:**
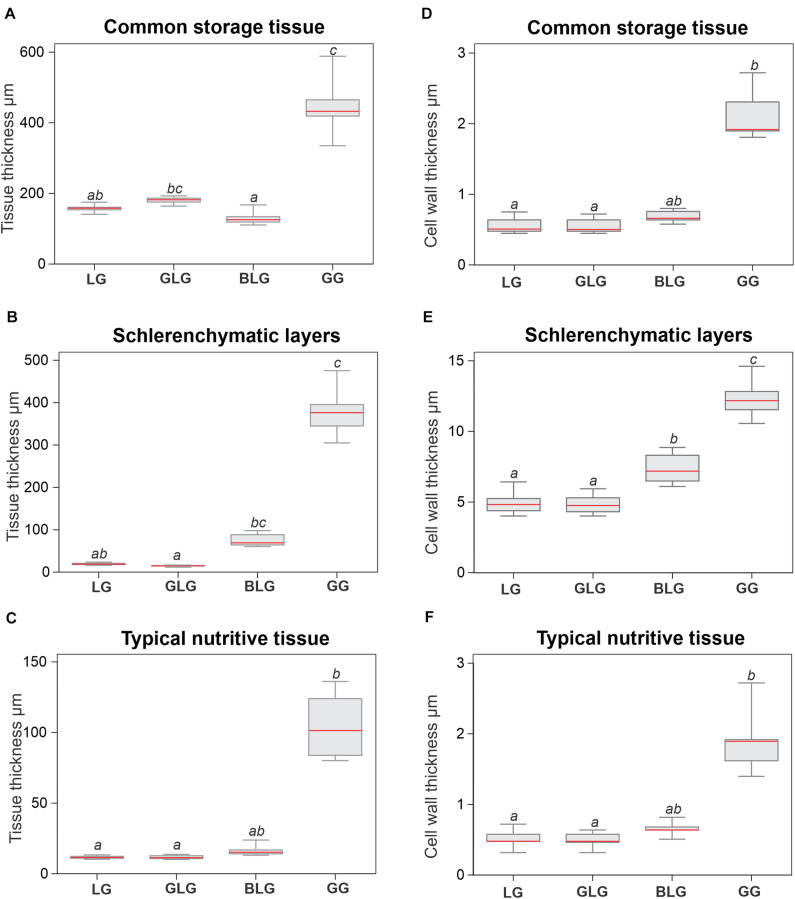
Histometry of the tissues and cell walls of the four *Lopesia* galls on *Mimosa gemmulata* Barneby (Fabaceae) pinna-rachis **(A–C)** Tissue thickness. **(D–F)** Cell wall thickness. BLG, brown lanceolate bivalve-shaped gall, GG, globoid bivalve-shaped gall, GLG, green lanceolate bivalve-shaped gall, LG, lenticular bivalve-shaped gall. *n* = 5 per gall systems (5 regions per section from 1 sections); *p* < 0.05. Same letters on bars indicate statistically equal values for the same variable, and different letters indicate different values.

In accordance with structural description, GG tissues are thicker than the tissues of the other three *Lopesia* galls (Figure 3). The GG common storage tissue is 177% thicker than that of the LG (*p* < 0.001), and 249% thicker than that of the BLG (*p* < 0.001), but there is no significant difference between the GG and the GLG regarding the thickness of the common storage tissue (Figure 3A). The GLG common storage tissue is 249% thicker than that of the BLG (*p* < 0.001). The GG schlerenchyma is 2,009% thicker than that of the LG (*p* < 0.001), and 2,495% thicker than that of the GLG (*p* < 0.001), but there is no significant difference between the GG and the BLG regarding the thickness of the schlerenchyma (Figure 3B). The BLG schlerenchyma is 348% thicker than that of the GLG (*p* < 0.001). There is no significant difference among the schlerenchyma thickness of the GL, the GLG and the BLG (Figure 3B). The GG typical nutritive tissue is 772% thicker than that of the LG (*p* < 0.001), and 813% thicker than that of the GLG (*p* < 0.001), but there is no significant difference between the GG and the BLG regarding the thickness of the typical nutritive tissue (Figure 3C). There is no significant difference of the typical nutritive tissue thickness among the LG, GLG, and BLG.

The cell walls of the schlerenchyma are thicker than the cell walls of the common storage and typical nutritive tissues on the four *Lopesia* galls (Figures 3D–F). The cell walls of the GG schlerenchyma are 141% thicker than the cell walls of the LG, 160% thicker than the cell walls of the GLG, and 76% thicker than the cell walls of the BLG (*p* < 0.001). The cell walls of the BLG schlerenchyma are 48% thicker than the cell walls of the GLG, and 37% thicker than the cell walls of the LG (*p* < 0.001). There is no significant statistical difference between the cell wall thickness of the schlerenchyma of the LG and GLG (*p* = 0.958). The cell walls of the GG storage and typical nutritive tissues are thicker than the cell walls of the GL and GLG (*p* < 0.001). There is no significant difference among in the cell wall thickness of the common storage tissues (Figure 3D) and typical nutritive tissues (Figure 3F) among the GL, GLG, and GBG.

#### Histochemical Profiles

**FIGURE 4:**
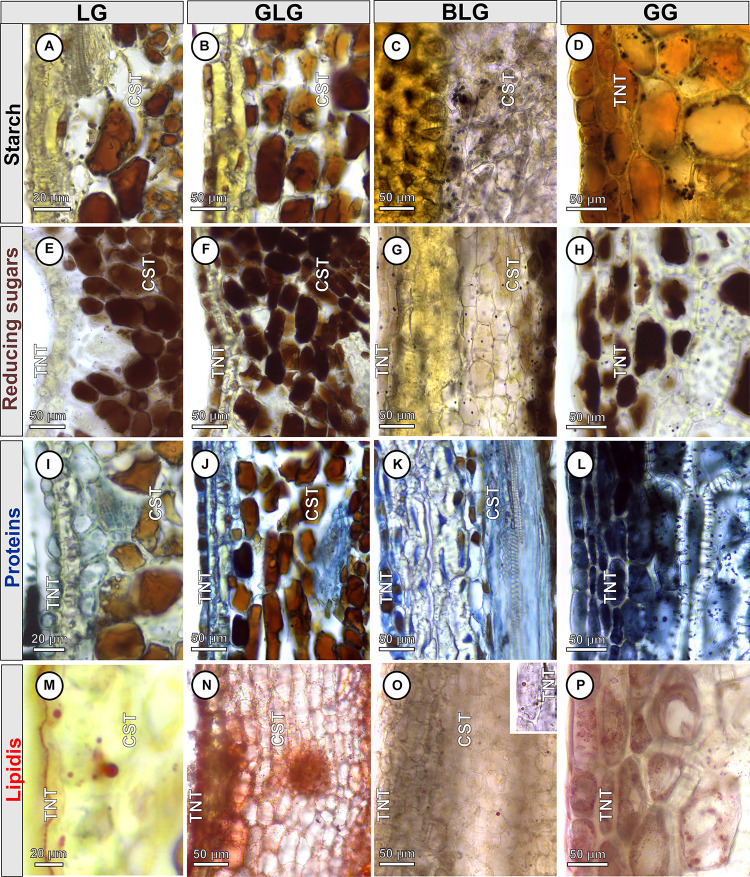
Histochemical profiles of the four *Lopesia* galls on *Mimosa gemmulata* Barneby (Fabaceae) pinna-rachis in transverse sections. **(A–D)** Starch grains stained in black. **(E–H)** Reducing sugars stained in brown. **(I–L)** Proteins stained in blue **(M–P)** Lipidic droplets stained in red. BLG, brown lanceolate bivalve-shaped gall; CST, common storage tissue; GG, globoid bivalve-shaped gall; GLG, green lanceolate bivalve-shaped gall; LG, lenticular bivalve-shaped gall; TNT, typical nutritive tissue.

Starch, reducing sugars, proteins, and lipids are detected both in the non-galled pinna-rachis and in the *Lopesia* galls (Table 1). Starch grains are detected only in the protoplasm of the common storage tissue of the LG (Figure 4A), GLG (Figure 4B), and BLG (Figure 4C). Starch grains (Figure 4D) are detected in the protoplasm of the GG common storage tissue, schlerenchyma and typical nutritive tissue. Reducing sugars are detected in the protoplasm of the common storage tissue and typical nutritive tissue of the LG (Figure 4E), GLG (Figure 4F), BLG (Figure 4G), and GG (Figure 4H). There is a centripetal gradient of reducing sugars in the LG, GLG, and BLG. In the GG, the reducing sugars accumulate in a bidirectional gradient. Proteins are detected in the protoplasm of the common storage tissue, schlerenchyma and typical nutritive tissue of the LG (Figure 4I), GLG (Figure 4J), BLG (Figure 4K), and GG (Figure 4L), forming a centripetal gradient toward the nutritive cells. Lipidic droplets are detected in the protoplasm of the common storage tissue and typical nutritive tissue of the LG (Figure 4M), GLG (Figure 4N), BLG (Figure 4O), and GG (Figure 4P).

**TABLE 1:** Histochemical profiles of the four *Lopesia* galls on *Mimosa gemmulata* (Fabaceae).

	**Outer compartment**								**Inner compartment**			
	**Common storage tissue**				**Schlerenchymatic layer**				**Typical nutritive tissue**			
	**LG**	**GLG**	**BLG**	**GG**	**LG**	**GLG**	**BLG**	**GG**	**LG**	**GLG**	**BLG**	**GG**
**Histochemistry**												
Starch	+	+	+	+	−	−	−	+		−	−	+
Reducing sugars	+	+	+	+	−	−	−	+	+	+	+	+
Proteins	+	+	+	+	−	−	+	+	+	+	+	+
Lipids	+	+	+	+	+	+	−	+	+	+	+	+

*BLG, brown lanceolate bivalve-shaped gall; LG, lenticular bivalve-shaped gall; GG, globoid bivalve-shaped gall; GLG, green lanceolate bivalve-shaped gall. The symbols indicate (**−**) absence and (+) presence.*

#### Immunocytochemical Profiles

**FIGURE 5:**
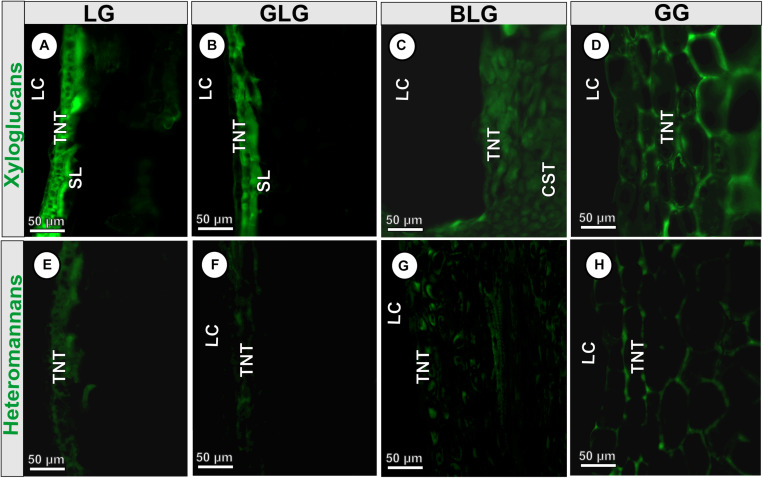
Immunocytochemical profiles of the four *Lopesia* galls on *Mimosa gemmulata* Barneby (Fabaceae) pinna-rachis in transverse sections. **(A–D)** Xyloglucans labeled by LM15. **(E–H)** Heteromannans labeled by LM20. BLG, brown lanceolate bivalve-shaped gall; CST, common storage tissue; GG, globoid bivalve-shaped gall; GLG, green lanceolate bivalve-shaped gall; LC, larval chamber; LG, lenticular bivalve-shaped gall; TNT, typical nutritive tissue; SL, schlerenchymatic layer.

The epitopes of xyloglucans recognized by LM15 are moderately labeled in the cell walls of the BLG (34 Gy) and GG (36.7 Gy) common storage tissue; intensely labeled in the cell walls of the LG schlerenchyma (63 Gy), and typical nutritive tissue (47.6 Gy) (Figure 5A); and moderately labeled in the cell walls of the GLG schlerenchyma (39.8 Gy), and weakly labeled in the cell walls of the typical nutritive tissue (17.26 Gy) (Figure 5B). The xyloglucans are moderately detected in the cell walls of the BLG typical nutritive tissue (38.6 Gy; Figure 5C), and intensely detected in the GG typical nutritive tissues (47.1 Gy; Figure 5D). The epitopes of heteromannans recognized by LM21 are weakly labeled in the cell walls of the typical nutritive tissue of the LG (20.6 Gy; Figure 5E), GLG (19.7 Gy; Figure 5F), BLG (12 Gy; Figure 5G), and moderately labeled in the cell walls of the GG typical nutritive tissue (31.6 Gy; Figure 5H and Table 2).

**TABLE 2:** Average of gray value and intensity of reaction of the epitopes for hemicelluloses in the tissues of the four *Lopesia* galls on *Mimosa gemmulata* (Fabaceae).

	**Outer compartment**								**Inner compartment**			
	**Common storage tissue**				**Schlerenchymatic layer**				**Typical nutritive tissue**			
	**LG**	**GLG**	**BLG**	**GG**	**LG**	**GLG**	**BLG**	**GG**	**LG**	**GLG**	**BLG**	**GG**
**Immunocytochemistry**												
Xyloglucans	0	0	34.0	36.7	63	39.8	0	0	47.6	17.2	38.6	47.1
Intensity	−	−	++	++	+++	++	−	−	+++	+	++	+++
Heteromannans	0	0	19.7	0	0	0	0	0	20.6	19.7	12	31.6
Intensity	−	−	+	−	−	−	−	−	+	+	+	++

*BLG, brown lanceolate bivalve-shaped gall; LG, lenticular bivalve-shaped gall; GG, globoid bivalve-shaped gall; GLG, green lanceolate bivalve-shaped gall.*

*Intensity of reaction: (**−**) negative (=0 Gy value); (+) weak (10–20 Gy value); (++) moderate (21–39.99 Gy value); (+++) intense (≥40 Gy value).*

## Discussion

**FIGURE 6:**
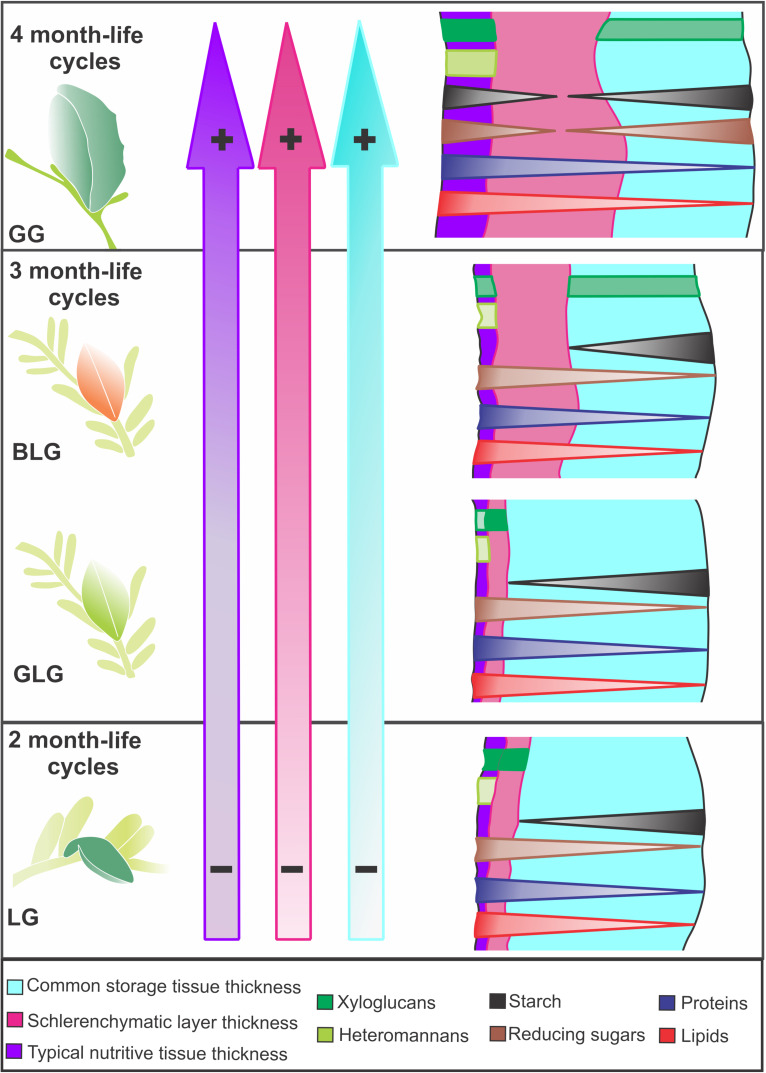
Diagrams of the structural and nutritional profiles of the four *Lopesia* galls on *Mimosa gemmulata* with their respective lifespans. The color gradients in the purple, pink and blue arrows, and the symbols (– and +) indicate tissue thickness investment. The diagrams of the transverse sections illustrate the gradients of the nutritive molecules in gall tissues. BLG, brown lanceolate bivalve-shaped gall; GG, globoid bivalve-shaped gall; GLG, green lanceolate bivalve-shaped gall; LG, lenticular bivalve-shaped gall.

The four *Lopesia* galls on *Mimosa gemmulata* have peculiarities regarding the investment in cell walls, tissue stratification, and accumulation of metabolites. The *Lopesia* species associated to the GG has the longest lifespan and distinct structural and nutritional profiles compared with the other three *Lopesia* galls (Figure 6). The GG has peculiar bidirectional gradients of starch and reducing sugars, which indicate additional substrates to the synthesis of xyloglucans and heteromannans in cell walls. In the typical nutritive tissues, the cell wall xyloglucans and heteromannans together with the protoplasm accumulated reducing sugars, proteins and lipids constitute the pool of energetic resources for the four galling *Lopesia*. Peculiarly, the GG with its 4 month-life cycle has the highest structural and nutritional investment in the storage and the nutritive tissues, and schlerenchymatic layers, which relates to the highest demand for gall development and galling insect establishment.

### Structural Peculiarities in the *Lopesia* Galls

Gall development and tissue compartmentalization depend on the galling insect behavior and how long the galling insect stimulus lasts (Bronner, 1992; Bragança et al., 2017; Oliveira et al., 2016). The duration of the gall cycle can determine the rate of cell divisions, elongation, and tissue complexity (Carneiro et al., 2017; Costa et al., 2018), and determine the structural profile of the storage tissues (Rohfritsch, 1992), which are distinct among the *Lopesia* galls. The GG with the 4-month life cycle has the highest stratification of the common storage tissue, when compared with the other gall morphospecies with the 2-month life cycle (LG) and 3-month life cycles (GLG and BLG) due both to higher hyperplasia and cell hypertrophy. In addition, the thicker cell walls of the GG common storage tissue indicate a distinct metabolic investment for the synthesis of cell wall components as cellulose, hemicellulose and pectins than observed in the other three *Lopesia* galls. This investment in cell wall thickness is important to control the turgor pressure during cell hypertrophy (Chanliaud et al., 2002), and confers an additional support for the GG. The structural and physiological traits of the common storage tissue have been related to the sink and storage of water and energetic resources (Castro et al., 2012; Oliveira et al., 2017; Bragança et al., 2020b). Water and energetic molecules accumulation increase gall succulence (tissue thickness) (Oliveira et al., 2017), and can also be consequence of the longest lifespan, as evidenced in the GG on *M. gemmulata* with the thickest cell walls as well as higher stratification of the common storage tissue.

The differentiation of the schlerenchymatic cell layers is associated with life cycle of the *Lopesia* galls on *M. gemmulata*. Secondary walls are mainly composed of cellulose, hemicelluloses (xylans and glucomannans), and lignins, whose biosynthesis is involved in the scavenging of free radicals (Liu et al., 2018; Zhong et al., 2019). The lignification process confers mechanical support and may also protect gall tissues against the high oxidative stress originated from the galling organism respiration and cell metabolism (Oliveira et al., 2010, 2011b, 2017; Isaias et al., 2015). The higher thickness of the GG cell walls indicates that the biosynthesis and deposition of the secondary wall components can be more expressive in galls with longer lifespans. The longest time the galling *Lopesia* associated to the GG remains inside the gall, and the higher impact of its breathing and feeding seem to result in the thick schlerenchymatic layers, which may favor the dissipation of oxidative stress and gall maintenance. Controversly, the LG and the GLG also have conspicuous schlerenchymatic layers, but with the thinnest cell walls amongst the *Lopesia* galls, while the BLG has an intermediate condition in cell wall and schlerenchymatic layer thickness among the four *Lopesia* galls on *M. gemmulata*. The differences in the duration of the life cycles even in 1–2 months relate to the thickness of the cell walls and the stratification of the schlerenchymatic layers on the *Lopesia* galls associated to *M. gemmulata*, which support the premise that the lignification process is triggered and supported by the galling insect activity and is more intense the longer the galling insect lifespan is.

The cells of the typical nutritive tissue develop due to the galling insect stimuli over the host plant tissues, as they are directly impacted by the nutrition of the inducers (Bronner, 1992). The cell walls and stratification of the typical nutritive tissues of the galls with 2- and the 3-month-life cycles (LG, BLG, and GLG) have similar thickness, which can indicate the low and constant nutritional demand of the three gall-inducing *Lopesia*. Differently, the primary cell walls and stratification of the typical nutritive tissue of the GG are thicker, which seems to be consequence of the greater feeding stimulus and high nutritional demand of the *Lopesia* larvae with the longest lifespan (4 month-life cycle).

### Nutritional Profiles in the Protoplasm of the *Lopesia* Galls

The life cycles did not alter the evaluated compounds linked to the nutritional profiles of the four *Lopesia* galls on *M. gemmulata*. However, the histochemical gradients of carbohydrates are bidirectional in the GG. The common storage tissues of four *Lopesia* galls are starch-rich, and, unexpectedly, starch grains also accumulate in the GG nutritive tissue. Starch is an insoluble polysaccharide that must be broken down in monosaccharides or disaccharides by invertases, such as D-glucose, activity (Morris and Arthur, 1984) in gall storage tissues (Oliveira et al., 2010, 2011a; Bragança et al., 2017). The inverse gradients of accumulation of starch and reducing sugars in the four *Lopesia* galls implies in the activity of enzymes and may result in adequate substrate for the biosynthesis of new cell wall components (Taylor, 2008; Polko and Joseph, 2019), such as xyloglucans (Pauly and Keegstra, 2016) and heteromannans (Voiniciuc et al., 2019) in *Lopesia* galls. These hemicelluloses are synthesized by Golgi-localized glycosyltransferases (GTs), and the resulting polysaccharides are secreted to the plant cell wall via exocytosis (Driouich et al., 2012; Voiniciuc et al., 2019). Accordingly, the bidirectional gradients of starch and reducing sugars in the common storage and typical nutritive tissues of the GG may be associated with additional substrates to biosynthesis of xyloglucans and heteromannans, and the composition of the primary cell walls (Pauly and Keegstra, 2016).

The proteins and lipids are energetic molecules that support the four gall-inducing *Lopesia* nutrition and gall structural maintenance. The proteins can act in the scavenging of free radicals and the maintenance of redox-potential homeostasis in tissues of plants (Meyer et al., 2019; Foyer et al., 2020) and of galls induced by different galling organisms (Schönrogge et al., 2000; Oliveira et al., 2010; Isaias et al., 2015; Silva et al., 2019). The protein accumulation in the nutritive cells of the *Lopesia* galls can have antioxidant function due to highest cellular metabolism in this tissue compartment. The accumulation of lipids in the storage, and/or typical nutritive tissue of Cecidomyiidae galls on different host plants are linked to the intrinsic metabolism of the host plants (Moura et al., 2008; Oliveira et al., 2010; Ferreira and Isaias, 2014; Amorim et al., 2017; Nogueira et al., 2018), as is true for *M. gemmulata*. The lipid droplets accumulated in the common storage tissue are related to the maintenance of the cellular machinery, while in the nutritive cells, they are food resources for the *Lopesia* galls. In the typical nutritive tissue, the lipids may become available by the activity of a lipase-like protein expressed in the salivary glands of Cecidomyiidae larvae during feeding, which can be involved in extra-oral digestion (Hatchett et al., 1990; Shukle et al., 2009; Al-Jbory et al., 2018).

### Nutritional Profiles in Cell Walls of *Lopesia* Galls

Xyloglucans and heteromannans are hemicelluloses involved in cell expansion and rigidity (Park and Cosgrove, 2015; Cosgrove, 2016, 2018; Voiniciuc et al., 2019), which also work out as reserve of carbohydrates accumulated in plant cell walls of some cotyledons, mainly in seeds of Fabaceae species (Buckeridge et al., 2000; Santos et al., 2004). Recently, the xyloglucans were reported as reserve carbohydrates in cell walls of nematode-induced galls on *Miconia* spp. (Melastomaceae) (Ferreira et al., 2020), and cell walls of the common storage and of the typical nutritive tissues of Cecidomyiidae-induced galls on *Inga ingoides* (Fabaceae) (Bragança et al., 2020a). Herein, the epitopes of xyloglucans labeled by LM15 in the common storage tissue of the BLG and the GG, and in the schlerenchymatic layers of the LG and GLG can influence the dynamics of cell wall expansion and rigidity. Both the xyloglucans and the heteromannans are detected in the primary cell walls of the typical nutritive tissue of the four *Lopesia* galls on *M. gemmulata*. While the overall labeling of heteromannans in the four *Lopesia* galls relates to structural function, the labeling of the xyloglucans in the typical nutritive tissues relates not only to the structural, but also to the nutritional function.

We demonstrated that the primary walls of the GG are thicker, and therefore a greater investment in the synthesis of xyloglucans as reserve carbohydrates occurs, which indicate the support for the galling *Lopesia* with the longest life cycle high nutritional demands. The xyloglucan monosaccharides can become available for the galling *Lopesia* nutrition by the activity of β-D-galactosidase and β-D-glucosidase, which may be found in salivary glands and/or midgut of Cecidomyiidae larvae (Grover et al., 1988). Even though the heteromannans and the xyloglucans are labeled in primary cell walls of the four *Lopesia* galls independently of their lifespans, the GG thickest cell walls and typical nutritive tissue indicate a higher support for the 4-month life cycle.

## Conclusion

The 1–2-month variation of *Lopesia* gall lifespans did not impact the type of energetic molecules, but the investment in cell walls and tissue stratification, especially regarding the common storage tissue. Moreover, cell walls have configured as sites of additional carbohydrate accumulation not only for structural, but for nutritional purposes. The bivalve-shaped globoid galls with the 4-month lifespan associated to *M. gemmulata* demanded the highest structural and nutritional support, as expected.

## Data Availability Statement

The raw data supporting the conclusions of this article will be made available by the authors, without undue reservation.

## Author Contributions

EC, DO, and RI designed the experiments, analyzed the data, and drafted the manuscript. EC and DF performed the experiments. All authors contributed to the article and approved the submitted version.

## Conflict of Interest

The authors declare that the research was conducted in the absence of any commercial or financial relationships that could be construed as a potential conflict of interest.

## Footnotes

1 http://rsb.info.nih.gov/ij

## References

[B1] Al-Jbory Z. Anderson K. M. Harris M. O. Mittapalli O. Whitworth R. J. Chen M. S. (2018). Transcriptomic analyses of secreted proteins from the salivary glands of wheat midge larvae. *J. Insect Sci.* 18:17. 10.1093/jisesa/iey009 31329739

[B2] Amorim D. O. Ferreira B. G. Fleury G. (2017). Plant potentialities determine anatomical and histochemical diversity in *Mikania glomerata* Spreng. galls. *Braz. J. Bot.* 40 517–527. 10.1007/s40415-016-0357-9

[B3] Bragança G. P. Oliveira D. C. Isaias R. M. S. (2017). Compartmentalization of metabolites and enzymatic mediation in nutritive cell of Cecidomyiidae galls on *Piper arboretum* Aubl. (Piperaceae). *J. Plant Stud.* 6 11–22. 10.5539/jps.v6n1p11

[B4] Bragança G. P. P. Alencar C. F. Freitas M. S. C. Isaias R. M. S. (2020a). Hemicelluloses and associated compounds determine gall functional traits. *Plant Biol.* 22 981–991. 10.1111/plb.13151 32597563

[B5] Bragança G. P. P. Freitas M. S. C. Isaias R. M. S. I. (2020b). The influence of gall position over xylem features in leaflets of *Inga ingoides* (Rich.) willd. (Fabaceae: Caesalpinioideae). *Trees* 35 199–209. 10.1007/s00468-020-02027-1

[B6] Bronner R. (1992). “The role of nutritive cells in the nutrition of cynipids and cecidomyiids,” in *Biology of Insect-Induced Galls*, eds Shorthouse J. D. Rohfritsch O. (New York, NY: Oxford University Press), 118–140.

[B7] Brundett M. C. Kendrick B. Peterson C. A. (1991). Efficient lipid staining in plant material with sudan red 7B or fluoral yellow 088 in polyethylene glycol–glycerol. *Biotech. Histochem.* 66 111–116. 10.3109/10520299109110562 1716161

[B8] Buckeridge M. S. Santos H. P. Tiné M. A. S. (2000). Mobilisation of storage cell wall polysaccharides in seeds. *Plant Physiol.* 38 141–156. 10.1016/S0981-9428(00)00162-5

[B9] Bukatsch F. (1972). Bermerkungen zur doppelfärbung astrablau-safranin. *Mikrokosmos* 61:255.

[B10] Carneiro R. G. S. Isaias R. M. S. Moreira A. S. F. P. Oliveira D. C. (2017). Reacquisition of new meristematic sites determines the development of a new organ, the Cecidomyiidae gall on *Copaifera langsdorffii* Desf. (Fabaceae). *Front. Plant Sci.* 8:1622. 10.3389/fpls.2017.01622 29033957 PMC5625070

[B11] Castro A. C. Oliveira D. C. Moreira A. S. F. P. Lemos-Filho J. P. Isaias R. M. S. (2012). Source-sink relationship and photosynthesis in the horn-shaped gall and its host plant *Copaifera langsdorffii* Desf. (Fabaceae). *S. Afr. J. Bot.* 83 121–126. 10.1016/j.sajb.2012.08.007

[B12] Chanliaud E. Burrows K. M. Jeronimidis G. Gidley M. J. (2002). Mechanical properties of primary plant cell wall analogues. *Planta* 215 989–996. 10.1007/s00425-002-0783-8 12355159

[B13] Chen X. Yang Z. Chen H. Qian Q. Liu J. Wang C. (2020). A complex nutrient exchange between a gall-forming aphid and its plant host. *Front. Plant Sci.* 11:811. 10.3389/fpls.2020.00811 32733495 PMC7358401

[B14] Cosgrove D. J. (2016). Plant cell wall extensibility: connecting plant cell growth with cell wall structure, mechanics, and the action of wall modifying enzymes. *J. Exp. Bot.* 67 463–476. 10.1093/jxb/erv511 26608646

[B15] Cosgrove D. J. (2018). Diffuse growth of plant cell walls. *Plant Physiol.* 176 16–27. 10.1104/pp.17.01541 29138349 PMC5761826

[B16] Costa E. C. Carneiro G. S. C. Silva-Santos J. Isaias R. M. S. (2018). Biology and development of galls induced by *Lopesia* sp. (Diptera: Cecidomyiidae) on leaves of *Mimosa gemmulata* (Leguminosae: Caesalpinioideae). *Aust. J. Bot.* 66 161–172. 10.1071/BT17099

[B17] Driouich A. Follet-Gueye M. L. Bemard S. Kousar S. Chevalier L. Vicré-Gibouin M. (2012). Golgi-mediated synthesis and secretion of matrix polysaccharides of the primary cell wall of higher plants. *Front. Plant Sci.* 3:79. 10.3389/fpls.2012.00079 22639665 PMC3355623

[B18] Ferreira B. G. Álvarez R. Avritzer S. C. Isaias R. M. S. (2017). Revisiting the histological patterns of storage tissues: beyond the limits of gall-inducing taxa. *Botany* 95 173–184. 10.1139/cjb-2016-0189

[B19] Ferreira B. G. Álvarez R. Bragança G. P. Alvarenga D. R. Pérez-Hidalgo N. Isaias R. M. S. (2019). Feeding and other gall facets: patterns and determinants in gall structure. *Bot. Rev.* 85 78–106. 10.1007/s12229-019-09207w

[B20] Ferreira B. G. Bragança G. P. Isaias R. M. S. (2020). Cytological attributes of storage tissues in nematode and eriophyid galls: pectin and hemicellulose functional insights. *Protoplasma* 257 229–244. 10.1007/s00709-019-01431-w 31410590

[B21] Ferreira B. G. Carneiro R. G. S. Isaias R. M. S. (2015). Multivesicular bodies differentiate exclusively in nutritive fast-dividing cells in *Marcetia taxifolia* galls. *Protoplasma* 252 1275–1283. 25613290 10.1007/s00709-015-0759-8

[B22] Ferreira B. G. Isaias R. M. S. (2014). Floral-like destiny induced by a galling Cecidomyiidae on the axillary buds of *Marcetia taxifolia* (Melastomataceae). *Flora* 209 391–400.

[B23] Formiga A. T. Silveira F. A. O. Fernandes G. W. Isaias R. M. S. (2014). Phenotypic plasticity and similarity among gall morphotypes on a superhost, *Baccharis reticularia* (Asteraceae). *Plant Biol.* 17 512–521. 25124804 10.1111/plb.12232

[B24] Foyer C. H. Baker A. Wright M. Sparkes I. A. Mhamdi A. Schippers J. H. M. (2020). On the move: redox-dependent protein relocation in plants. *J. Exp. Bot.* 71 620–631. 10.1093/jxb/erz330 31421053

[B25] Gonçalves S. J. M. R. Isaias R. M. S. Vale F. H. A. Fernandes G. W. (2005). Sexual dimorphism of *Pseudotectococcus rolliniae* Hodgson & Gonçalves 2004 (Hemiptera Coccoidea Eriococcidae) influences gall morphology on *Rollinia laurifolia* Schltdl. (Annonaceae). *Trop. Zool.* 18 161–169. 10.1080/03946975.2005.10531219

[B26] Grover P. B. JR. Ross D. R. Shukle R. H. (1988). Identification and partial characterization of digestive carbohydrases in larvae of the Hessian fly, *Mayetiola destructor* (Say) (Diptera: Cecidomyiidae). *Arch. Insect Biochem. Physiol.* 5, 59–72. 10.1002/arch.940080106

[B27] Hatchett J. H. Kreitner G. L. Elzinga R. J. (1990). Larval mouthparts and feeding mechanism of the Hessian fly (Diptera: Cecidomyiidae). *Ann. Entomol. Soc. Am.* 83 1137–1147.

[B28] Isaias R. M. S. Oliveira D. C. Moreira A. S. F. P. Soares G. L. G. Carneiro R. G. S. (2015). The imbalance of redox homeostasis in arthropod-induced plant galls: mechanisms of stress generation and dissipation. *Biochim. Biophys. Acta* 1850 1509–1517. 10.1016/j.bbagen.2015.03.007 25813551

[B29] Johansen D. A. (1940). *Plant Microtechnique.* New York, NY: McGraw-Hill Book.

[B30] Jorge N. C. Souza-Silva E. A. Alvarenga D. R. Saboia G. Soares G. L. G. Zini C. A. (2018). Structural and chemical profiles of *Myrcia splendens* (Myrtaceae) leaves under the influence of the galling *Nexothrips* sp. (Thysanoptera). *Front. Plant Sci.* 9:1521. 10.3389/fpls.2018.01521 30459785 PMC6232307

[B31] Karnovsky M. J. (1965). A formaldehyde-glutaraldehyde fixative of high osmolarity for use in electron microscopy. *J. Cell Biol.* 27 137–138.

[B32] Kraus J. E. Arduin M. (1997). *Manual Básico de Métodos em Morfologia Vegetal.* Rio de Janeiro: Editora da Universidade Rural.

[B33] Liu Q. Luo L. Zheng L. (2018). Lignins: biosynthesis and biological functions in plants. *Int. J. Mol. Sci.* 19:335. 10.3390/ijms19020335 29364145 PMC5855557

[B34] Mani M. S. (1964). *Ecology of Plant Galls.* The Hague: Dr. W. Junk Publishers. 10.1007/978-94-017-6230-4

[B35] Marcus S. E. Blake A. W. Benians T. A. S. Lee K. J. D. Poyser C. Donaldson L. (2010). Restricted access of proteins to mannan polysaccharides in intact plant cell walls. *Plant J.* 61 191–203. 10.1111/j.1365-313X.2010.04319.x 20659281

[B36] Marcus S. E. Verhertbruggen Y. Herve C. Ordaz-Ortiz J. J. Farkas V. Pedersen H. L. (2008). Pectic homogalacturonan masks abundant sets of xyloglucan epitopes in plant cell walls. *BMC Plant Biol.* 8:60. 10.1186/1471-2229-8-60 18498625 PMC2409341

[B37] Mazia D. Brewer P. A. Alfert M. (1953). The cytochemistry staining and measurement of protein with mercuric bromophenol blue. *Biol. Bull.* 104 57–67.

[B38] Meyer A. J. Riemer J. Rouhier N. (2019). Oxidative protein folding: state-of-the-art and current avenues of research in plants. *New Phytol.* 221 1230–1246. 10.1111/nph.15436 30230547

[B39] Meyer J. Maresquelle H. J. (1983). *Anatomie des Galles.* Berlin: Gerbrüder Borntrager.

[B40] Morris D. A. Arthur E. D. (1984). Invertase and auxin-induced elongation in internodal segments of *Phaseolus vulgaris*. *Phytochemistry* 23 2163–2167.

[B41] Moura M. Z. D. Soares G. L. G. Isaias R. M. S. (2008). Species-specific changes in tissue morphogenesis induced by two arthropod leaf gallers in *Lantana camara* L. (Verbenaceae). *Aust. J. Bot.* 56 153–160. 10.1071/BT07131

[B42] Nogueira R. M. Costa E. C. C. Santos-Silva J. Isaias R. M. S. (2018). Structural and histochemical profile of *Lopesia* sp. Rübsaamen. 1908 pinnula galls on *Mimosa tenuiflora* (Willd.) Poir. in a Caatinga environment. *Hoehnea* 45 231–239. 10.1590/2236-8906-80/2017

[B43] Oliveira D. C. Carneiro R. G. S. Magalhães T. A. Isaias R. M. S. (2011a). Cytological and histochemical gradients on two *Copaifera langsdorffii* Desf. (Fabaceae) – Cecidomyiidae gall systems. *Protoplasma* 248 829–837. 10.1007/s00709-010-0258-x 21207084

[B44] Oliveira D. C. Isaias R. M. S. Fernandes G. W. Ferreira B. G. Carneiro R. G. S. Fuzaro L. (2016). Manipulation of host plant cells and tissues by gall-inducing insects and adaptive strategies used by different feeding guilds. *J. Insect Physiol.* 84 103–113. 10.1016/j.jinsphys.2015.11.012 26620152

[B45] Oliveira D. C. Isaias R. M. S. Moreira A. S. F. P. Magalhães T. A. Lemos-Filho J. P. (2011b). Is the oxidative stress caused by *Aspidosperma* spp. galls capable of altering leaf photosynthesis? *Plant Sci.* 180 489–495. 10.1016/j.plantsci.2010.11.005 21421396

[B46] Oliveira D. C. Magalhães T. A. Carneiro R. G. S. Alvim M. N. Isaias R. M. S. (2010). Do Cecidomyiidae galls of *Aspidosperma spruceanum* (Apocynaceae) fit the pre-established cytological and histochemical patterns? *Protoplasma* 242 81–93. 10.1007/s00709-010-0128-6 20306094

[B47] Oliveira D. C. Moreira A. S. F. P. Isaias R. M. S. Martini V. Rezende U. C. (2017). Sink status and photosynthetic rate of the leaflet galls induced by *Bystracoccus matayba* (Eriococcidae) on *Matayba guianensis* (Sapindaceae). *Front. Plant Sci.* 8:1249. 10.3389/fpls.2017.01249 28791033 PMC5522869

[B48] Paiva J. G. A. Fank-de-Carvalho S. M. Magalhães M. P. Graciano-Ribeiro D. (2006). Verniz vitral incolor 500^®^ : uma alternativa de meio de montagem economicamente viável. *Acta Bot. Bras.* 20 257–264. 10.1590/S0102-33062006000200002

[B49] Park Y. B. Cosgrove D. J. (2015). Xyloglucan and its interactions with other components of the growing cell wall. *Plant Cell Physiol.* 56 180–194. 10.1093/pcp/pcu204 25613914

[B50] Pauly M. Keegstra K. (2016). Biosynthesis of the plant cell wall matrix polysaccharide xyloglucan. *Annu. Rev. Plant Biol.* 67 235–259. 10.1146/annurev-arplant-043015-112222 26927904

[B51] Polko J. K. Joseph J. K. (2019). The regulation of cellulose biosynthesis in plants. *Plant Cell* 31 282–296. 10.1105/tpc.18.00760 PMC6447023 30647077

[B52] Rezende U. C. Custódio J. F. Kuster V. C. Gonçalves L. A. Oliveira D. C. (2019). How the activity of natural enemies changes the structure and metabolism of the nutritive tissue in galls? Evidence from the *Palaeomystella oligophaga* (Lepidoptera) –*Macairea radula* (Metastomataceae) system. *Protoplasma* 256 669–677. 30446812 10.1007/s00709-018-1321-2

[B53] Rohfritsch O. (1992). “Patterns in gall development,” in *Biology of Insect-Induced Galls*, eds Shorthouse J. D. Rohfritsch O. (New York, NY: Oxford University Press), 60–86.

[B54] Rübsaamen E. H. (1908). Beitrage zur Kenntnis aussereuropaischer Zoocecidien. III. Beitrag: Gallen aus Brasilien und Peru. *Marcellia* 7, 15–79.

[B55] Santos H. P. Purgatto E. Mercier H. Buckeridge M. S. (2004). The control of storage xyloglucan mobilization in cotyledons of *Hymenaea courbaril*. *Plant Physiol.* 135 287–299. 10.1104/pp.104.040220 15133152 PMC429377

[B56] Sass J. E. (1951). *Botanical Microtechnique.* Ames, IA: Iowa State College Press.

[B57] Schönrogge K. Harper L. J. Lichtenstein C. P. (2000). The protein content of tissues in cynipid galls (Hymenoptera: Cynipidae): similarities between cynipid galls and seeds. *Plant Cell Environ.* 23 215–222. 10.1046/j.1365-3040.2000.00543.x

[B58] Shukle R. H. Mittapalli O. Morton P. K. Chen M. S. (2009). Characterization and expression analysis of a gene encoding a secreted lipase-like protein expressed in the salivary glands of the larval Hessian fly, *Mayetiola destructor* (Say). *J. Insect Physiol.* 55 105–112. 10.1016/j.jinsphys.2008.10.008 19026654

[B59] Silva A. F. D. M. Kuster V. C. Rezende U. C. Oliveira D. C. (2019). The early developmental stages of gall-inducing insects define final gall structural and histochemical profiles: the case of *Bystracoccus mataybae* galls on *Matayba guianensis*. *Botany* 97 427–438. 10.1139/cjb-2019-0017

[B60] Taylor N. G. (2008). Cellulose biosynthesis and deposition in higher plants. *New Phytol.* 178 239–252. 18298430 10.1111/j.1469-8137.2008.02385.x

[B61] Voiniciuc C. Dama M. Gawenda N. Stritt F. Pauly M. (2019). Mechanistic insights from plant heteromannan synthesis in yeast. *Proc. Natl. Acad. Sci. U.S.A.* 116 522–527. 10.1073/pnas.1814003116 30584101 PMC6329948

[B62] Zhong R. Cui D. Ye Z.-H. (2019). Secondary cell wall biosynthesis. *New Phytol.* 221 1703–1723. 10.1111/nph.15537 30312479

